# Making the Case for Addressing Health Disparities: What Drives Providers and Payers?

**DOI:** 10.1089/heq.2017.0034

**Published:** 2018-05-01

**Authors:** Margaret Johnson, Heather McPheron, Rachel Dolin, Julia Doherty, Lisa Green

**Affiliations:** ^1^ L&M Policy Research, LLC, Washington, District of Columbia.; ^2^ David A. Winston Health Policy Fellowship, Washington, District of Columbia.

**Keywords:** business case, case studies, disparities, economic case, health disparities

## Abstract

**Purpose:** The creation of the Centers for Medicare & Medicaid Services Office of Minority Health placed increased emphasis on federal efforts to address health disparities. Although the literature establishes a social justice case for addressing health disparities, there is limited evidence of this case being sufficient for businesses to invest in such initiatives. The purpose of this study was to better understand the “business case” behind an organization's investment in health disparity reduction work.

**Methods:** We conducted six case studies (44 on-site interviews) with diverse private-sector provider and payer organizations.

**Results:** While providers and payers cited business rationales for initiating disparity-focused efforts, their motivations differed.

**Conclusion:** As federal entities address health disparities, and payment models shift from volume to value, engaging private stakeholders with the leverage to move the health disparities needle is of principal importance.

## Introduction

Health disparities have ramifications for the entire population, regardless of the community to which they accrue.^1–3^ The business benefits for organizations positioned to address these disparities—such as payers and providers—are not always clear in a predominantly fee-for-service environment.^4,5^ The movement toward value-based payment methods presents an opportunity for policymakers to align the implicit social justice case with business motivations for reducing disparities.

The marriage of social justice with economics can be viewed through business cases. In the context of reducing health disparities, a *business case* is an organization's rationale for investing in a socially responsible action that also promises financial return within a reasonable time frame through cost reductions, increased revenues, or both.^6–9^ In addition to direct cost savings or revenue generated, these efforts can also yield a return for the investing entity in the form of improvements in service delivery, marketing capacity, or sustainability.^5–8^ Thus, a compelling business case would encourage organizations to invest time, effort, and funds in an initiative to reduce health disparities.^6^

Accordingly, the objective of this study was to conduct a series of case studies with private-sector providers and payers to better understand their business cases. We sought to learn what motivated these stakeholders to engage in this work and glean their lessons learned. Ultimately, these case studies could provide insights to private-sector entities and federal policymakers on how to invest in programs aimed at reducing health disparities.

### Conceptual model

**FIG. 1.**
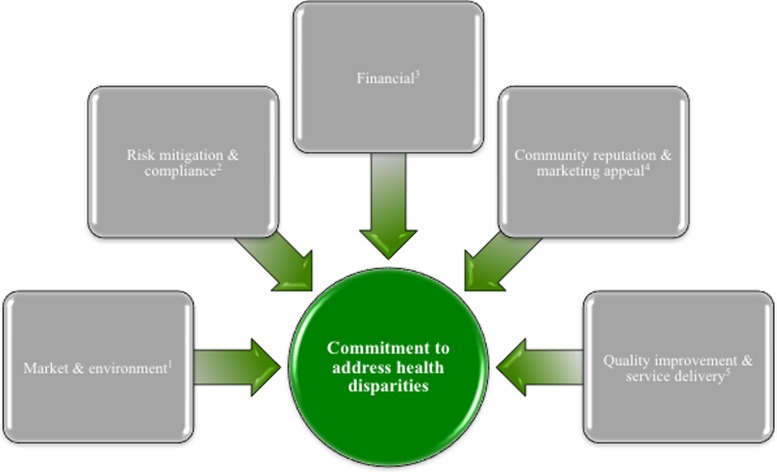
Primary Motivators for Commitment to Address Health Disparities. Source: Adapted from Environmental Scan and Annual Summary Report submitted to CMS OMH under contract HHSM-500-2011-00019I/HHSM-500-T0005.^14,15^ Notes: (1) Market & environment—External conditions and/or pressures influence an organization's pursuit of initiatives aimed at reducing health disparities; (2) Risk mitigation & compliance—Issues of compliance and risk mitigation (e.g., corrective action plans, legal action) are influencing factors on the organization's health disparity reduction activities; (3) Financial—Organization expects financial factors (e.g., enhanced reimbursement, decreased costs) to be affected by its health disparity reduction activities; (4) Community reputation & marketing appeal—Reputation and/or marketing appeal are drivers underlying the organization's health disparity reduction activities; (5) Quality improvement & service delivery—Aspects of quality and service delivery (e.g., Healthcare Effectiveness Data and Information Set measures, patient outcomes) are considerations the organization seeks to address through its health disparity reduction activities.

We conducted an environmental scan encompassing a literature review and key informant interviews with health equity and disparity experts from academia and industry to inform our conceptual model. This scan illuminated the domains of a health disparity business case (see Fig. 1). These domains served as an organizing framework for the case study interview protocols and for analyzing results.

## Methods

We conducted case studies through site visits to providers and payers—two stakeholder groups positioned to reduce health disparities given their interactions with patients. The goal of the site visits was to gather information on the motivations underlying each organization's decision to engage in activities to reduce health disparities, its experiences in designing and implementing these activities, and its challenges in sustaining this work. Internal Review Board (IRB) approval was not required by the Federal sponsor; additionally, we believe this study would have been exempt as the interviews were voluntary and uncompensated, and interviewees were asked only about topics associated with their routine job activities. Interviewees were fully informed of how the information would be used. No sensitive personal information was collected.

### Sample selection and recruitment

To identify candidates, we conducted web searches for U.S.-based organizations that had won awards or were profiled for their commitment to addressing health disparities; this yielded 73 organizations. We ranked organizations on how well their program(s) aligned with the objectives of our study, which narrowed the list to 36. To further refine the list, we conducted brief semistructured calls with candidates to confirm our understanding of their efforts to build a business case to address health disparities and gauge interest in participation. From the refined list of 18, we invited six organizations—those that described a comprehensive longstanding program and/or more than one initiative—to participate in a voluntary and uncompensated site visit.

### Data collection

We developed semistructured discussion guides (one each for providers and payers), integrating domains from the conceptual framework (Fig. 1), and elements of Chin et al.'s disparities roadmap.^10^ We structured questions around: the business case development process, incentives to invest in disparity reduction initiatives, concerns related to implementation and sustainability, and lessons learned.^10^

Two senior researchers, who led the discussions, and one research assistant, who took transcript-style notes, conducted each site visit. Some interviews were conducted in small groups while others were one-on-one.

### Data analysis

We used Dedoose, a web-based relational database, to store, code, and analyze the interview notes.^11^ We developed a code tree that employed a 30-code hierarchical scheme and mirrored the discussion guides and domains from our conceptual model (see Fig. 1).^12^ For each code, we developed a definition, inclusion, and exclusion criteria, and coding guidance. For each set of notes, one researcher coded the notes and another reviewed them; we required agreement from both researchers on the final coding. The team completed a structured content analysis using a hybrid inductive–deductive coding approach.^13^ Additionally, the team prepared a written summary of each individual case study; each organization confirmed factual accuracy of the associated summary.

## Results

Between March and May 2016, we completed a total of 44 on-site discussions with 93 individuals at three provider and three payer organizations. Across the six case studies, we interviewed persons key to the development, implementation, and ongoing operations of health disparity reduction initiatives, including chief executive officers, chief financial officers, quality improvement staff, health disparity initiative leaders, physicians, and clinicians, among others; see Table 1.

**Table 1.** Case Study Organization Profiles

Name	Tax status	Institution type/product lines	No of interviews and interviewees	Service region	Location
**Stakeholder group: providers**					
FHM	Nonprofit	Community hospital	7 interviews, 9 individuals	East	Frederick, Maryland
Methodist Healthcare	Nonprofit	Faith-based integrated delivery system	6 interviews, 17 individuals	South	Memphis, Tennessee
VFC	Nonprofit	Safety net health system/community health clinic	7 interviews, 9 individuals	West	Los Angeles, California
**Stakeholder group: payers**					
HPHC	Nonprofit	Medicare, Medicaid, Commercial products	8 interviews, 13 individuals	East	Wellesley, Massachusetts
Health Net	For profit	Medicare, Medicaid, Commercial products	6 interviews, 30 individuals	West	Woodland Hills, California
Highmark, Inc.	Nonprofit	Medicare, Medicaid, Commercial products	10 interviews, 15 individuals	East/ Midwest	Pittsburgh, Pennsylvania

*Source: Authors' analysis of self-reported data provided by case study organizations and publicly available information, collected in Spring, 2016.*^15^

FHM, Frederick Memorial Hospital; Methodist Healthcare, Methodist Le Bonheur Healthcare; HPHC, Harvard Pilgrim Health Care; VFC, Venice Family Clinic.

For providers and payers, the business case to reduce health disparities is a driving motivator in their decision about whether and how to invest in health disparity reduction activities. Because the business case manifests differently between these stakeholders, we present these findings separately. We summarize findings by first presenting primary influences on stakeholders' decisions to invest in reducing health disparities (“origin stories”). Second, we present the stakeholders' motivations for investing in addressing health disparities. Finally, we present challenges faced in sustaining their efforts.

### Providers

#### Origin story

All three provider organizations launched their health disparity-focused initiatives to address a perceived need among underserved and/or vulnerable individuals in their respective service areas. The prioritized communities included individuals with constrained resources, those experiencing homelessness, members of the Deaf community, and immigrants.

To address the needs of these populations, each of the organizations developed tailored initiatives to mitigate health disparities. Table 2 presents examples of some of these organizations' initiatives. To meet the unique needs of their constituents, these initiatives vary in breadth and depth; some reflect a population health-based approach, whereas others focus on specific health conditions and outcomes.

**Table 2.** Examples of Provider Initiatives Implemented to Address Health Disparities

Name	Initiatives
FMH	Interpreter Services: In the early 1990s, FMH instituted an American Sign Language interpreter services program to meet the needs of a large local deaf population. In the past 25 years, FMH's in-person interpreter services program has grown significantly in staff and now includes Spanish language interpreters, as well as interpreting services through telephone and remote video feed.
	Population Health: FMH recently established a senior management position to focus on ensuring that new health disparities' reduction efforts align with the organization's strategy to improve the area population's health. Examples of newly formed initiatives include a program to increase connections between FMH and the community, improving access to prenatal care for uninsured and underinsured mothers in the community, opening a clinic for chronically ill patients, and providing dental services for uninsured community residents.
Methodist Healthcare	CHN: Launched in 2006, Methodist, in partnership with a core group of churches in the adjacent community, created the CHN program as a means to develop trust and relationships with community members aimed at improving population health and reducing inappropriate use of health services. As part of CHN, leaders in the faith community agree to participate in the program by signing a “covenant,” which is a commitment to participate in CHN that requires the churches to identify volunteers within their respective congregations who serve as community liaisons to Methodist navigators.
VFC	Diabetes Care Management Program: The program began in 2014 to assist patients with management of their diabetes with the goals of reducing medical complications and avoiding hospitalizations. Primary care physicians refer patients with elevated Hemoglobin A1c to the program, through which they meet regularly with nurses who track the patients' progress in controlling their blood glucose levels and provide ongoing support and referrals to health education services.
	Health Education Department: The goal of the department is to empower patients with the knowledge and tools to make healthy decisions for themselves to ultimately reduce disparities. The department's primary program is one-on-one educational counseling, wherein health educators meet with the provider and the patient to work collaboratively to tailor health maintenance education to the needs of the individual patient. The department also conducts community outreach with various nonhealthcare organizations to gauge what services would be valuable to the community.

*Source: Authors' analysis of self-reported data provided by case study organizations, Spring 2016.*^15^

CHN, Congregational Health Network.

#### Motivations

While these organizations may have launched their work for similar reasons, each had different motivations for ongoing commitment to the initiatives. All providers cited at least two of the motivators shown in Figure 1. The most commonly discussed motivator was market/environment, followed by community reputation and marketing appeal (see Fig. 1 for a complete definition). The other motivators were only noted by one of the providers, and thus are not discussed in detail in this article.

Leadership at all three provider organizations cited the environment in which they operate, coupled with market demands, as major drivers of their commitment to address health disparities. More specifically, one provider suggested its involvement in addressing health disparities as a selling point to its supporters and donors. Disparity reduction efforts help the provider uphold its commitment, both financially and operationally, to delivering services to any person in their community. The second provider cares for a large portion of the low-income and uninsured population in its community, and addressing health disparities is a priority from both a business and community impact standpoint. The third provider proactively reacted to changing demographics in its market by establishing an interpreter services program designed to mitigate and avoid potential health disparities stemming from poor patient–provider communication. All three providers also highlighted community engagement as a central aspect of their respective organizations' approach to combating disparities.

Two organizations were also motivated to address health disparities to maintain their reputation as leaders in providing health services to all individuals who need care, regardless of ability to pay. Each organization has earned this reputation through its years of commitment to improving access to care for vulnerable populations. One provider discussed the importance of upholding its mission and reputation within the community for providing access to care for the poor and homeless—a mission the organization established at its outset. Years later, the organization remains committed to this social justice motivation, which today aligns with its formal recognition as a safety net provider. The other provider also focuses on ensuring access, as it is the only health system that operates facilities throughout all areas of the city it serves and by maintaining contracts with all payers that offer coverage in the region.

#### Overcoming barriers and sustainability

While all the providers discussed robust commitment to their health disparities work, they also acknowledged difficulties sustaining and advancing their efforts. The organizations cited two challenges: (1) maintaining sufficient funding to preserve the initiatives addressing disparities, and (2) engaging internal and external stakeholders to establish support for the initiatives and to generate human and financial resources. Although different, these challenges are inseparable and overlapping: Providers seek the engagement of stakeholders to identify funds or human resources to sustain their health disparities work—yet continuing to identify resources also requires significant time and effort from internal stakeholders. Two participant organizations said they struggle more with maintaining engagement of internal stakeholders than external stakeholders. To mitigate this challenge, one provider said it focuses on engaging departments supporting its disparities' initiatives, while the other continuously seeks support—sharing updates and progress reports—from leadership to ensure financial resources are allocated to sustain existing activities. In both cases, the sustainability of initiatives depends on internal support.

### Payers

#### Origin story

All three payers described a drive to prioritize the needs of their evolving member populations as the impetus for their entrées into health disparities work. In two cases, these efforts originated in siloes on a limited scale led by individual staff champions until management determined it was worth investing in broader efforts. The third payer experienced a change in its operating structure, which dramatically shifted the composition of its member population and negatively affected the payer's quality scores. Opportunity to improve these scores spurred the payer's investment in disparity reduction initiatives.

Table 3 provides examples of select initiatives each payer organization implemented to address health disparities identified within its respective member populations. The initiatives vary and reflect differences in how each payer tailored interventions to its population's needs.

**Table 3.** Examples of Payer Initiatives to Address Health Disparities

Name	Initiatives
HPHC	Culture of Diversity & Inclusion. After assessing its organizational readiness to change, HPHC made structural changes to elevate its commitment to developing an organization-wide diversity and inclusion strategy. The plan created the Center for Inclusion Initiatives in 2012 and appointed a director to oversee the center, who leads activities that support progress toward integrating elements of diversity and inclusion into each aspect of the organization's strategic plan and business practices.
	Transgender-Inclusive Care Benefits. In response to an employer group customer's request, HPHC rolled out transgender-inclusive care benefits in 2010 to a limited group of employer clients. The benefit has since been offered more broadly as part of a strategy to offer it to all HPHC members and in response to a 2014 Massachusetts state mandate.
Health Net	Childhood Immunization Status Combination-3. Through a combination of provider collaboration initiatives, community educational programs, and a Russian-language media campaign, Health Net, is working to increase vaccination compliance rates across the state of California with a specific focus on the Russian-speaking community in Sacramento County, as immunization rates are particularly low within this community.
	Low-Income Health Disparities and D-SNPs. Health Net developed a series of targeted interventions to reduce readmission rates and close gaps in care for all members enrolled in its D-SNP plans.
Highmark	RELE Data Collection. Beginning in 2005, Highmark started collecting RELE data elements from its internal systems to identify gaps in care in particular communities. Interventions initiated in response to these analyses include activities to improve rates of immunizations, preventive services, glaucoma screenings, and diabetes screenings.
	Faith-Based Learning Collaborative. Highmark initiated a Faith-Based Learning Collaborative in 2011 after meeting with respected church leaders and social service agencies in Southwestern Pennsylvania that serve primarily African American communities with high prevalence of chronic conditions and where cardiovascular disease is a leading cause of death. Working closely with church leaders to understand their priorities, Highmark supports the community's interest in addressing heart health through a jointly designed learning collaborative called, “Take Care of My Heart.”

*Source: Authors' analysis of self-reported data provided by case study organizations, Spring 2016.*^15^

D-SNPs, Dual-Eligible Special Need Plans; RELE, Race, Ethnicity, Language, and Education.

#### Motivations

Similar to their origin stories, each payer discussed similar motivations for their commitment to addressing health disparities. All payers cited both financial and quality improvement/service rationales for addressing health disparities (see Fig. 1 for definitions). In addition, two of the three payers discussed community reputation, fostering community engagement, and marketing appeal as key factors underlying their commitment.

All three payers identified financial pressures as influencing their decision to address health disparities, specifically citing customer retention or increasing membership as motivators. Revenue preservation, which is inextricably linked to quality and service pressures due to financial incentive structures, is also a key driver. In addition, payers said addressing health disparities allows them to shed more light on, and, in some cases, improve quality measures.

Finally, two of the three payers said their commitment to addressing health disparities stems from a desire to enhance their community reputation and marketing appeal. One payer described its efforts to achieve the National Committee for Quality Assurance Multicultural Healthcare accreditation, which, interviewees said, solidified the organization's reputation among health insurers as an early adopter of standards for providing culturally competent care. Another payer described product offerings created in response to employer groups' requests to address a specific health disparity. This same payer has also developed offerings to engage its existing and increasingly diverse membership and to attract new members; for example, it developed an insurance product to cover non-U.S.-based family members visiting relatives in the United States.

#### Overcoming barriers and sustainability

Despite commitment to their initiatives, payers face challenges sustaining these efforts. While demonstrating return on investment (ROI) is a top priority for ensuring sustainability of their health disparities work, none of the payers can currently measure it. One payer explained that associations can be detected between its interventions and outcomes, but there are too many factors at play to demonstrate causality. Two of the payers have implemented more than one disparity reduction initiative simultaneously, making it difficult to isolate the effect of a given intervention.

Additionally, all three payers emphasized that identifying opportunities to address disparities, planning, and implementing interventions requires interdepartmental collaboration, which can be challenging. To foster engagement among internal stakeholders, two organizations described how leaders continually find ways to communicate the relevance of disparity interventions to keep the work a priority within the business.

## Discussion

This study gathered information on the motivations underlying healthcare stakeholders' decisions to invest in initiatives to reduce health disparities. In turn, this work sought to expand the evidence base of strategies organizations employ to design, implement, and sustain these efforts. The case studies revealed that business case motivations are a driving factor in organizations' decisions to invest in and maintain such work, despite challenges with quantifying an ROI. The business case manifests differently between providers and payers, however.

Although case study organizations' original impetus for addressing health disparities varied, we observed differences by stakeholder group. All providers focused on how best to improve the delivery of care and enhance access to services, citing community reputation and marketing appeal and/or market as factors influencing their decision to address health disparities. Each of the payers identified both financial and quality improvement/service—where quality improvement leads to enhanced reimbursement—as key considerations underlying their commitment to address health disparities. In all case studies, these motivators were not mutually exclusive.

For both stakeholder audiences, sustainability health disparity-focused interventions depend on an organization's willingness to allocate resources—a decision that is challenging in the face of limited resources. Providers' rationale for continuing to devote resources to such work was nuanced—but the role of resources was nonetheless apparent. Their programs are sustained by a desire to deliver care to underserved and vulnerable individuals who would likely come to them for care regardless. Engaging internal or external stakeholders, coupled with identifying resources needed to operationalize the work, are the foremost challenges to sustaining provider-based interventions. For payers, the business case for reducing health disparities was framed in a traditional light, in which payers spoke about the importance of demonstrating the financial benefit of investments and achieving an ROI. Key challenges include building measure-based evidence to evaluate progress, quantifying the financial impact of specific initiatives, and maintaining internal support among staff.

Ultimately, for providers and payers, continued investment in these initiatives is predicated on demonstrating value. For all organizations we studied, this depends on: (1) engaging internal and external stakeholders to leverage and extend resources, and (2) collecting data to measure outcomes. Providers focus their efforts on improving outcomes tied to ROI through financial incentives that reward quality; in contrast, payers' efforts to collect data are directly tied to trying to measure ROI. While organizations report that measuring ROI is a priority, external pressure would make the business case more compelling. Enhanced reimbursement for reporting quality measures stratified by selected variables or the offer of implementation support for disparity reduction interventions may encourage mainstream adoption of the business case.

### Limitations

Our study has several limitations. First, due to the purposive sample, the findings presented here may not be generalizable. Second, our approach to identifying potential respondents was limited by the availability of public data; it is plausible that qualified organizations were not considered due to an absence of information. The sample was limited due to resource constraints; and finally, our recruitment process relied on voluntary participation. While our findings are based on a limited sample, they nonetheless offer important insights: we ensured representation from different regions, a variety of organizational structures, and we spoke with 93 individuals. Future research could include additional case studies to further illuminate the value proposition for healthcare stakeholders to engage in disparity reduction efforts.

### Policy implications

This study offers encouraging examples of stakeholders developing business cases to support health disparity reduction activities. To address health disparities on a broader scale, increased engagement by stakeholders in health disparity reduction efforts is necessary.

Prioritizing policy changes that focus on reducing health disparities is vital. Policymakers can appeal to stakeholders by presenting this work as an avenue to reduce costs and improve quality, while protecting revenue. Elevating the importance of addressing health disparities necessitates policymakers consider a multipronged effort to spur stakeholder engagement, including: (1) instituting data-gathering requirements to measure the presence of health disparities; and (2) creating incentives to reduce disparities in addition to rewarding quality. With value-based payment arrangements on the rise, organizations' willingness to invest in initiatives to reduce disparities as a lever to control costs and/or improve quality may grow organically; however, policy changes may provide an accelerant.

## Abbreviations Used

CHN: Congregational Health Network
D-SNPs: Dual-Eligible Special Need Plans
FMH: Frederick Memorial Hospital
HPHC: Harvard Pilgrim Health Care
IRB: Internal Review Board
Methodist Healthcare: Methodist Le Bonheur Healthcare
RELE: Race, ethnicity, language, and education
ROI: Return on investment
VFC: Venice Family Clinic

## References

[1] LaVeist TA, Gaskin DJ, Richard P The economic burden of health inequalities in the United States. Washington, D.C.: Joint Center for Political and Economic Studies, 2009

[2] Brott A, Dougherty A, Williams S, et al. The economic burden shouldered by public and private entities as a consequence of health disparities between men and women. Am J Mens Health. 2011;5:528–539 22064880 10.1177/1557988311421214

[3] Alberti P, Bonham A, Kirch D Making equity a value in value-based health care. Acad Med. 2013;88:1619–1623 24072123 10.1097/ACM.0b013e3182a7f76f

[4] Braveman PA, Kumanyika S, Fielding J, et al. Health disparities and health equity: the issue is justice. Am J Public Health. 2011;101(S1):S149–S155 21551385 10.2105/AJPH.2010.300062 PMC3222512

[5] Ruger JP Ethics of the social determinants of health. Lancet. 2004;364:1092–1097 15380971 10.1016/S0140-6736(04)17067-0 PMC3988689

[6] Leatherman S, Berwick D, Iles D, et al. The business case for quality: case studies and an analysis. Health Aff (Millwood). 2003;22:17–30 12674405 10.1377/hlthaff.22.2.17

[7] Bovbjerg RR, Hatry HP, Morley E Making a business case for reducing racial and ethnic disparities in healthcare: Key issues and observations. Washington, D.C.: Urban Institute, 2009

[8] Kilpatrick KE, Lohr KN, Leatherman S, et al. The insufficiency of evidence to establish the business case for quality. Int J Qual Health Care. 2005;17:347–355 15788462 10.1093/intqhc/mzi034

[9] Greene SB, Reiter KL, Kilpatrick KE, et al. Searching for a business case for quality in Medicaid managed care. Health Care Manage Rev. 2008;33:350–360 18815500 10.1097/01.HCM.0000318772.59771.b2

[10] Chin MH, Clarke AR, Nocon RS, et al. A roadmap and best practices for organizations to reduce racial and ethnic disparities in health care. J Gen Intern Med. 2012;27:992–1000 22798211 10.1007/s11606-012-2082-9 PMC3403142

[11] Dedoose, Version 7.5.9 [computer program]. Los Angeles, CA: SocioCultural Research Consultants, LLC, 2016

[12] King N Template Analysis. In: Symon G, Cassel C, eds. Qualitative Data Analysis in Organisational Research: A Practical Guide. London: Sage, 1998

[13] Waring T, Wainwright D Issues and challenges in the use of template analysis: two comparative case studies from the field. The Electronic Journal of Business Research Methods. 2008;6:85–94

[14] McPheron H, Phung Q, Doherty J, et al. Environmental Scan for CMS OMH. Baltimore, MD: Centers for Medicare & Medicaid Services (Unpublished report); April 2015

[15] McPheron H, Johnson M, Dolin R, et al. Annual Summary Report for CMS OMH. Baltimore, MD: Centers for Medicare & Medicaid Services (Unpublished report), July 2016

